# How do the non-cognitive skills affect retirees’ reemployment? Evidence from China

**DOI:** 10.3389/fpubh.2023.1128241

**Published:** 2023-12-19

**Authors:** Haiyan Jia, Xiaoyu Sai, Hongyun Si, Jinming Wang

**Affiliations:** ^1^School of Public Administration and Policy, Shandong University of Finance and Economics, Jinan, China; ^2^School of Sociology and Ethnology, University of Chinese Academy of Social Sciences (UCASS), Beijing, China

**Keywords:** non-cognitive skills, retirees, reemployment, social capital, human capital

## Abstract

**Introduction:**

Promoting the reemployment of retirees is important to effectively recognize the capacity of older adults and to help governments cope with an aging global population. Existing research on the factors that impact reemployment has mainly focused on the role of traditional forms of human capital, like education and experience, while ignoring non-cognitive skills.

**Methods:**

Based on 3,693 samples, this study examines the impact of non-cognitive skills on the reemployment of Chinese retirees using the Logit model through the lens of human capital theory.

**Results:**

The results show that non-cognitive skills incentivize retirees to seek reemployment. The incentive effect is greater for retirees who are male, live in a rural household, and are of lower age and education level. Further, the mediation effect model reveals the mediating role of social capital between non-cognitive skills and the reemployment of retirees. Social capital is important to the promotion of retiree reemployment.

**Discussion:**

This study ultimately sheds light on the relationship between non-cognitive skills and the reemployment of retirees. Findings will help improve governments’ understandings of non-cognitive skills so that they may develop better policies on retiree reemployment.

## 1 Introduction

The global population will reach 9.7 billion in 2050, with the number of individuals over 65 years old exceeding 1.5 billion. The older adults will account for 16% of the total population (1). The already significant increase in the aging population has incurred a series of issues such as insufficient labor supply, rising dependency ratio, as well as increasing government welfare and pension expenditures (2). The deterioration of mental health, social isolation, and poverty have also become increasingly prominent among the older adults (3, 4). In response to these challenges, the World Health Organization launched the Active Ageing Initiative in 2002 to encourage older people to return to the workforce (5). Despite reaching retirement age, most healthy older people still have a strong work capacity and a high willingness to participate in the labor market. Data released by the German Federal Statistical Office show that the labor force participation rate of the older adults aged 60 to 64 was 63.6% in 2021 (6); meanwhile, Japan’s 2021 Edition of the White Papers on Aging Society, released by the Japanese government, notes that the employment rate of individuals aged 60 to 64 in 2020 was 71.0% (7). Therefore, it is important for employment policies to effectively recognize the capacity of older adults and to promote the reemployment of the retirees.

Successful reemployment post-retirement depends largely on the level of human capital possessed by the older adults (8). An intangible form of capital in which an individual can continuously invest, and human capital is naturally cumulative (9). However, the decline in physical functions such as memory and stamina that is associated with increasing age, as well as a lack of learning opportunities and vocational training, can also lead to a sustained depreciation of human capital. In the labor market, older adults usually encounter discrimination from employers and are precariously employed (10). Therefore, academic research has increasingly focused on how to effectively develop the human capital of the older adults. The literature usually divides human capital into two categories when examining the relationship between the level of human capital of retirees and reemployment. One is traditional human capital, such as education, health, experience, and skills. The more educated and healthier the older adults are, the stronger their willingness to extend their working years and the higher the probability of their reemployment (11). With the development of human capital theory, scholars have realized that traditional human capital focuses only on cognitive skills, while non-cognitive skills such as self-esteem, self-confidence, and ambition have an equally important impact on workers’ reemployment (12). However, existing research on the impact of retirees’ non-cognitive skills on reemployment is insufficient, in particular due to the lack of heterogeneous research on different retirees. This is not conducive to the development of their human capital and to the formulation of reemployment policies. The objective of this paper is thus to develop and test the relationship between non-cognitive skills and the reemployment of chinese retirees. We test the hypotheses pertaining to these, what we term the effect of non-cognitive skills on post-retirement reemployment and the intermediary role of social capital, using secondary data collected from 3,693 Retirees in China.

In 2000, China became an aging society. The growth rate of the aging population is higher than that of other countries (13). The effective management of the older adults in the workforce, the nation’s labor shortage, and the challenges that are incurred by an aging population has become an important issue for China’s sustainable economic and social development. Research on this issue will benefit China and may also help improve the situation in other countries. For this study, we used data from the China Family Panel Studies (CFPS) in 2018 to construct a composite indicator to measure the non-cognitive skills of retirees. Using a logit econometric model, we conducted an empirical study on the impact of non-cognitive skills on reemployment after retirement. Subsequently, we used social capital as the intermediary variable to test influence.

This study contributes to research in several ways. Firstly, this is the first study on the impact of retirees’ non-cognitive skills on reemployment, thus the findings help advance research on human capital. Secondly, by designing the measurement indicators of the Big Five Inventory (BFI), we have developed a new theoretical framework that can provide an important reference for identifying and measuring the non-cognitive skills of retirees. Thirdly, the study offers insight on the influential mechanism of non-cognitive skills on the reemployment of retirees, on group differences, and on the intermediary role of social capital. The targeted practical suggestions may help inform employment policies in other countries around the world.

## 2 Literature review and research hypotheses

### 2.1 Non-cognitive skills and reemployment after retirement

The concept of non-cognitive skills was first proposed by Bowles and Gintis (14). They classified skills into cognitive and non-cognitive categories. Cognitive skills are mainly related to intelligence and comprise learning, calculating, reading, as well as logical thinking. Non-cognitive skills are mainly related to personality and psychological factors such as emotions, self-esteem, and beliefs. They can be described as “relatively enduring, automatic patterns of thoughts, feelings, and behaviors that people exhibit in similar situations across time” (15). The main methods to measure non-cognitive skills include personality tests, questionnaires, and behavioral experiments. BFI is an internationally recognized measurement scale for questionnaires. We adopted this scale to measure the non-cognitive skills of the participants in this study.

Non-cognitive skills are mainly determined by factors such as family background and education. It has a significant impact on an individual’s social life, in particular, their work performance. Laible and Brenzel (16) asserted that non-cognitive skills are important predictors of workers’ performances in the labor market, and can significantly explain the income differences between workers. It can affect laborer’s occupation preferences, stimulate entrepreneurial motivation, and facilitate business decisions (17). Ultimately, non-cognitive skills can complement cognitive skills.

Research suggests that the personality of retirees affects job-seeking behavior and can effectively help predict their reemployment behavior. Individuals with unstable emotions and negative thoughts are less likely to find or maintain a job, while those with a positive self-image are more likely to be employed (18). Liu et al. (19) conducted a survey on 65 Chinese retired older adults and found that job-search self-efficacy significantly positively predicted reemployment willingness. Thus, it may be that improving the non-cognitive skills of retirees, such as encouraging extroverted and creative attitudes, may be effective in stimulating their initiative to re-enter the labor market. This study proposes the following hypothesis:

*H1*: Improving the non-cognitive skills of retirees will play a positive role in their reemployment.

### 2.2 Social capital and reemployment after retirement

The concept of social capital was proposed by Bourdieu (20) and it refers to the assets that are formed through individuals’ interpersonal relationships. It is grounded in the social connection established and maintained by individuals. The more social capital an individual possesses, the more active their job-seeking behavior. For retirees, the support of spouses, family members, and friends is important, particularly as increased social support makes them more likely to choose reemployment after retirement.

In addition, social capital can help people find information and resources related to the job market as well as access career networks, which is conducive to reemployment (21). A study revealed that one-third of jobs in Sweden were found through contacts (22). The older adults in the study who returned to the labor market had a higher number of contacts with more prestigious jobs. Their job search methods were mainly interpersonal and the rate of individuals who found work via their social networks was high (23).

Thus, it appears that social capital provides job seekers with a wider range of contacts connected to the labor market, who may disseminate information about job opportunities. Further, it can help individuals obtain material assistance and emotional support as they seek work (17). Social capital is therefore an important factor in promoting the reemployment of retirees. This study proposes the following hypothesis:

*H2*: Social capital has a significant positive effect on the reemployment of retirees.

### 2.3 Non-cognitive skills, social capital, and reemployment after retirement

Zheng et al. (24) claimed that non-cognitive skills have a significant predictive effect on social capital. Osler et al. (25) also explored how non-cognitive skills impact social capital. They found that people with stronger non-cognitive skills can better expand their social capital by building interpersonal networks and effectively communicating with others, resulting in a more favorable social status.

Research suggests that people with higher non-cognitive skills are more willing to actively participate in social activities and more likely to establish social ties with people who may promote their career development. People with extensive social networks are more likely to use their social capital as opposed to government-led services when job hunting and are more likely to be reemployed (26). This study proposes the following hypothesis:

*H3*: Social capital plays an intermediary role between non-cognitive skills and the reemployment of retirees, that is, non-cognitive skills can significantly increase the social capital of retirees and thus promote their reemployment.

**Figure 1:**
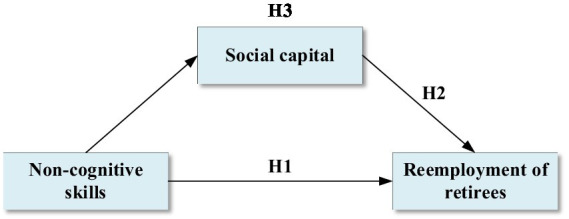
Theoretical framework of non-cognitive skills influencing the reemployment of retirees.

In conclusion, this study explores how non-cognitive skills and social capital impact the reemployment of retirees. This relationship is illustrated in Figure 1.

## 3 Materials and methods

### 3.1 Data source and sample composition

Data used in this study are from the CFPS project produced in 2018 (CFPS data are freely available at http://www.isss.pku.edu.cn/cfps/). CFPS is a nation-wide, longitudinal social survey project initiated by the Institute of Social Science Survey of Peking University. All participants provided informed consent. Ethical approval for data collection of all the CFPS waves was obtained from the Biomedical Ethics Committee of Peking University (approval number: IRB00001052-14010). CFPS is a multi-stage probability sample extracted by the implicit strategy, covering 25 provinces/cities/autonomous regions in China, and the target sample size is 16,000 households. The respondents include all family members in the sample households. The survey requested basic personal information such as income, work, health status and retirement status. The CFPS project is considered highly representative of the nation’s population, with its high-quality, micro data set that includes a full range of variables, and a large sample size from urban and rural areas across China. It provides authentic and reliable data for academic research and public policy analysis in various social sciences fields. For example, Ju et al. (27) used this data successfully to investigate the association between long-term exposure to major components of PM2.5 and worsening depressive symptoms, while Shi et al. (28) attained relevant results when employing the data to investigate the influencing factors of internet usage and its impact on well-being.

We processed the raw data as follows. First, individuals under 60 years of age and who were not yet retired were excluded from the sample. Second, to reduce the risk of biasing the experimental results, individuals with missing core variables and important information were also removed from this study. After integration, the data for 3,693 individuals were finally collected. Detailed socio-demographic statistics are shown in Table 1.

**Table 1:** Socio-demographic statistics in 2018.

Characteristic	Demographic data	Frequency	%
Gender	Male	2,052	55.56
	Female	1,641	44.44
Age (years)	65 and below	1,391	37.67
	66–70	1,176	31.84
	71–75	698	18.90
	76 and above	428	11.59
Education	No schooling	1,520	41.16
	Primary school	875	23.69
	Junior high school	785	21.26
	Senior high school and above	513	13.89
Marital status	Married	3,640	98.56
	Unmarried	53	1.44
Household registration	Rural	2,232	60.44
	Urban	1,461	39.56
Number of children	0–1	1,130	30.60
	2	1,274	34.50
	3 and above	1,289	34.90

### 3.2 Empirical model

In this study, the corresponding model estimation method was selected based on the type of dependent variable. The dependent variable is whether the older adults are reemployed after retirement, which is a binary choice variable. 1 indicates that the older adults are reemployed after retirement and 0 means that they are not. We used the Logit model for empirical analysis. The model (1) was set as follows:


(1) $$\text{Wor}k_{i}=\alpha_{1}+\beta_{1}{\text{Noncognitive}}_{i}+\gamma X_{i}+\varepsilon_{i}$$

$\text{Wor}k_{i}$ indicates whether they are reemployed or not; $\alpha_{1}$ is the constant term; ${\text{Noncognitive}}_{i}$ indicates the core explanatory variable, i.e., non-cognitive skills; $X_{i}$ indicates other control variables; and $\varepsilon_{i}$ indicates the random error term.

### 3.3 Variables and measurement

#### 3.3.1 Outcome variable

The outcome variable is the reemployment of retirees. First, we kept surveys where participants answered that they were retired (29). Then, within the remaining sample, if the “current work status” indicated activity, we considered that they chose reemployment after retirement (30). We assigned a value of 1 in this case; otherwise, it was 0. A total of 3,693 retirees were screened in this study, of which 1830 were considered reemployed. The reemployment rate is 49.55%.

#### 3.3.2 Treatment variable

The treatment variable is non-cognitive skills. This study referred to the BFI to define and measure the non-cognitive skills of retirees. The scale is a conceptual method for measuring an individual’s personality, which is not affected by specific situations (31). The BFI has good credibility and reliability, and it has been effectively verified by the World Bank and by researchers from countries such as Germany, Poland, and the UK (32). The scale classifies non-cognitive skills into five categories: extraversion, agreeableness, openness, conscientiousness, and emotional stability (33). Detailed explanations are shown in Appendix A.

The non-cognitive skills measurement items provided by CFPS are from the formal, simplified BFI. We referred to Nikčević et al. (34), and based on the relevant data from the CFPS (2018) project, we designed 15 indicators for the five dimensions described above. This design can effectively overcome the issue with the incomplete or arbitrary selection of indicators that was identified in previous studies.

Previous studies typically tested the measurement models by examining their reliability and construct validity (35, 36). Firstly, due to the large number of indicators involved, we used the variance inflation factor (VIF) to perform a multicollinearity test. The multicollinearity test results are reported in Table 2. The VIF values range from 1.11 to 1.42, with an average VIF of 1.24; and all VIF values are below 5, indicating no significant collinearity between variables (37). Secondly, we used the Cronbach’s alpha to report the current reliability of BFI in the Chinese. We found that the scale reliability coefficient is 0.6762. As suggested by Hair et al. (38), when scale reliability coefficient exceeds 0.5, the measurement model can be accepted. Finally, the Pearson Correlation Coefficient test was conducted for the 15 indicators in this study to prove the construct validity. The correlation coefficients between most of the indicators are significant. The results are shown in Appendix B, which demonstrates that we use BFI to measure the non-cognitive skills of Chinese retirees is effective.

In this study, the non-cognitive skills of individuals were determined by calculating the average after summation. The measurement results show that the mean value of the non-cognitive skills in the sample is 3.502. The mean value for each dimension is as follows: extraversion (3.425), agreeableness (3.905), openness dimension (3.096), conscientiousness (3.959), and emotional stability (3.123).

**Table 2:** Multicollinearity test results.

Five dimensions	Secondary indicators	VIF	1/VIF
Extraversion	Outgoing and sociable	1.34	0.748
	Talkative	1.23	0.815
	Reserved and conservative (reversed)	1.11	0.905
Agreeableness	Considerate of others	1.24	0.806
	Tolerant by nature	1.25	0.802
	Rude to others (reversed)	1.13	0.881
Openness	Originality	1.31	0.763
	Attach importance to artistic and esthetic experience	1.28	0.781
	Rich imagination	1.42	0.703
Conscientiousness	Efficient	1.32	0.756
	Rigorous and serious	1.16	0.862
	Often lazy (reversed)	1.16	0.861
Emotional stability	Often worried (reversed)	1.23	0.813
	Easily nervous (reversed)	1.24	0.805
	Copes well with stress	1.18	0.845
Mean VIF		1.24	

#### 3.3.3 Intermediary variable

The intermediary variable is social capital. In China, the expenditure of funds for interpersonal relationships is common, as it is part of a long-standing cultural tradition and the Chinese code of conduct. It is an important way for Chinese society to maintain interpersonal communications and social networks. In general, the more that is spent on an interpersonal relationship, the more extensive a family’s social interactions are likely to be and thus the more social capital it possesses. Referring to Zhao and Jiang‘s (39)study, we selected the previous year’s household expenditure on interpersonal relationships of their sample as the proxy variable for their social capital. The results of this study show that the mean value of retiree’s annual household expenditure on interpersonal relationships is 4355.779 yuan. The ratio of annual household expenditure on interpersonal relationships to household disposable income is 12.66% for all participants in the sample. The ratio for the retirees who are considered reemployed (16.39%) is significantly higher than those who are not (10.45%).

#### 3.3.4 Covariates

According to research practice, other relevant control variables were included in the model (40). For instance, regional macroeconomic variables were used, including the level of economic development, infrastructure, and urbanization, as well ratio of tertiary industry to secondary industry, degree of openness to the outside world, government expenditure on social security and employment, and population aging and urban unemployment rates (Data from China Statistical Yearbook).^1^ Second, social security characteristics variables were also employed, for example, by verifying for participation in endowment insurance and medical insurance. Third, household characteristics variables were included, such as the number of children and the retirement status of spouses. Fourth, individual characteristic variables were used: region, gender, age, level of education, marriage, household registration, cognitive skills, pension income, and number of years in the workforce. In addition, we controlled the fixed effects of different age groups in the employment market because of the significant differences in the living and working abilities of the older adults at different ages. The specific definitions and descriptive statistics of each variable are shown in Table 3.

**Table 3:** Descriptive statistics for variables used (*N* = 3,693).

Variables			Definition	Mean	SD	Range
Outcome variable	Reemployment of Retirees		Yes = 1; no = 0	0.496	0.500	[0,1]
Treatment variable	Non-cognitive skills		Mean value of all indicators after addition	3.502	0.416	[1.933,5]
Intermediary variable	Logarithm of the annual household expenditure on interpersonal relationships		Logarithm of the annual household expenditure on interpersonal relationships	6.926	2.846	[0,11.29]
Individual characteristic variables	Region		Province	36.778	14.566	[11,62]
	Gender		Male = 1; Female = 0	0.556	0.497	[0,1]
	Age		Unit: year	68.152	5.718	[60,93]
	Square of age		Square of age	4677.412	805.726	[3,600,8,649]
	Education level		No schooling = 1; primary school = 2; junior high school = 3; high school = 4; college degree or above = 5	2.11	1.152	[1,5]
	Marriage		Marriage = 1; not married = 0	0.986	0.119	[0,1]
	Household registration		Rural = 1; urban = 0	0.604	0.489	[0,1]
	Cognitive skills	Math test score	Composite score generated by math test	6.029	4.127	[0,23]
		Phrase test score	Composite score generated by word test	14.635	10.468	[0,34]
	Logarithm of pension income		Logarithm of monthly pension income	5.277	2.783	[0,9.473]
	Working years		Unit: year	43.9	1.347	35
Household characteristics variables	Number of children		Number of children	2.202	1.255	[0,9]
	Spouse retirement		Yes = 1; no = 0	0.66	0.474	[0,1]
Social security characteristics variables	Medical insurance		Yes = 1; no = 0	0.943	0.233	[0,1]
	Endowment insurance		Yes = 1; no = 0	0.727	0.446	[0,1]
Regional macroeconomic variables	Economic development level		Regional gross domestic product *per capita*	11.021	0.652	[10.353,11.851]
	Ratio of tertiary industry to secondary industry		Ratio of added value of the tertiary industry to that of the secondary industry	1.435	0.513	[0.86,4.348]
	Infrastructure level		Logarithm of road mileage	11.751	0.985	[9.481,12.712]
	Urbanization level		Logarithm of population density	8.033	0.348	[7.035,8.608]
	Degree of openness to the outside world		Proportion of foreign direct investment in gross domestic product	16.813	0.217	[0.027,67.139]
	Government social security and employment expenditure		Proportion of government social security and employment expenditure in gross domestic product	3.618	0.049	[1.308,6.259]
	Population aging		Ratio of older adults population to working age population	17.478	3.070	[11.04,22.69]
	Urban unemployment rate		Rate of registered unemployment in urban areas	3.225	0.470	[1.4,4]

### 3.4 Research design

**Figure 2:**
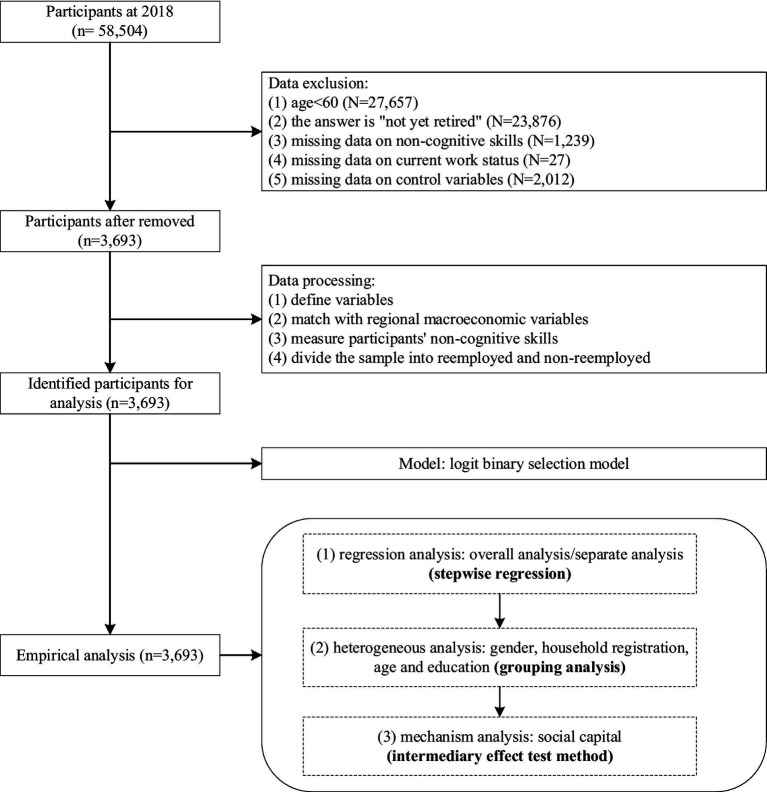
A flowchart of data analysis.

This is a study based on CFPS in 2018. The data analysis workflow is shown in Figure 2, including data exclusion, data processing and empirical analysis. Firstly, this study deleted samples under 60 years old and not yet retired, and the core variables such as non-cognitive skills, work status and control variables with missing values and unreasonable samples were eliminated. Secondly, this study redefined and assigned the variables, and matched the individual variables with the regional macroeconomic variables according to the China Statistical Yearbook, so as to supplement the data from the macro level. Finally, the empirical analysis of this study is carried out, including regression analysis, heterogeneity analysis and mechanism analysis.

## 4 Results

### 4.1 Regression analysis

#### 4.1.1 Overall analysis

The regression results are indicated in Table 4. Column (1) reflects the regression results when individual characteristic variables are added. Findings demonstrate that non-cognitive skills have a significant positive impact on the reemployment of retirees, with an impact coefficient of 0.381. The higher the non-cognitive skills of retirees, the greater the possibility of re-entering the labor market. This result remains true after adding the variables related to household characteristics, social security characteristics, and regional macroeconomic (see columns 2–4).

**Table 4:** Logit regression of the impact of non-cognitive skills on the reemployment of retirees.

			(1)	(2)	(3)	(4)	(5)
Variables			Reemployment of retirees				
Treatment variables	Non-cognitive skills		0.381***	0.366***	0.378***	0.355***	0.354***
			(0.099)	(0.106)	(0.103)	(0.096)	(0.099)
			[0.187, 0.575]	[0.158, 0.575]	[0.175, 0.581]	[0.166, 0.544]	[0.161, 0.547]
Individual characteristic variables	Gender		0.987***	0.950***	0.965***	0.943***	0.946***
			(0.076)	(0.079)	(0.079)	(0.084)	(0.083)
	Age		0.277	0.361*	0.325	0.267	0.192
			(0.187)	(0.208)	(0.206)	(0.191)	(0.268)
	Square of age		−0.003**	−0.004**	−0.003**	−0.003**	−0.002
			(0.001)	(0.002)	(0.002)	(0.001)	(0.002)
	Education level		−0.224***	−0.228***	−0.225***	−0.224***	−0.226***
			(0.074)	(0.076)	(0.074)	(0.071)	(0.073)
	Marriage		0.628	0.642	0.650	0.656	0.652
			(0.454)	(0.456)	(0.456)	(0.475)	(0.478)
	Household registration		1.823***	1.713***	1.512***	1.430***	1.431***
			(0.171)	(0.169)	(0.193)	(0.204)	(0.202)
	Cognitive skills	Math test score	0.079	0.087	0.087	0.110	0.109
			(0.066)	(0.070)	(0.069)	(0.078)	(0.078)
		Phrase test score	−0.161*	−0.153*	−0.135	−0.114	−0.113
			(0.082)	(0.084)	(0.083)	(0.090)	(0.089)
	Logarithm of pension income		−0.098***	−0.091***	−0.156***	−0.147***	−0.147***
			(0.025)	(0.024)	(0.034)	(0.028)	(0.028)
	Working years		−0.021	−0.023	−0.036	−0.018	−0.018
			(0.047)	(0.047)	(0.046)	(0.046)	(0.047)
Household characteristics variables	Number of children			0.104**	0.098**	0.083**	0.082**
				(0.045)	(0.045)	(0.0398)	(0.040)
	Spouse retirement			−0.295***	−0.310***	−0.255**	−0.252**
				(0.106)	(0.104)	(0.122)	(0.121)
Social security characteristics variables	Medical insurance				0.266	0.183	0.182
					(0.192)	(0.212)	(0.212)
	Endowment insurance				0.537***	0.473***	0.472***
					(0.183)	(0.170)	(0.169)
Regional macroeconomic variables	Economic development level					−0.556***	−0.558***
						(0.140)	(0.140)
	Ratio of tertiary industry to secondary industry					−0.679***	−0.679***
						(0.256)	(0.257)
	Infrastructure level					0.562***	0.566***
						(0.133)	(0.133)
	Urbanization level					0.069	0.069
						(0.131)	(0.130)
	Degree of openness to the outside world					2.526***	2.541***
						(0.722)	(0.721)
	Social security and employment expenditure					−4.108***	−4.126***
						(1.268)	(1.256)
	Population aging					0.085***	0.085***
						(0.021)	(0.021)
	Urban unemployment rate					−0.025	−0.027
						(0.191)	(0.190)
The fixed effects of different age groups			No	No	No	No	Yes
Pseudo R^2^			0.2457	0.2497	0.2544	0.2656	0.2658
Observations			3,693	3,693	3,693	3,693	3,693

The standard errors of clustering robustness at the provincial level are shown in parentheses, and 95% confidence interval in square brackets. ∗*p* < 0.05, ∗∗*p* < 0.01, ∗∗∗*p* < 0.001.

Column (5) shows the addition of all control variables, with consideration of the fixed effects of different age groups. The results show that the coefficient of non-cognitive skills is 0.354, which is significantly positive at the level of 1%. The goodness of fit (R^2^) indicates a good model fit and an overall significant model relationship. The positive effect of non-cognitive skills on the reemployment of retirees is confirmed by the above regression. H1 is therefore validated. In addition, most of the control variables are significant, which proves that the research hypothesis is set correctly, the variable selection is reasonable, and the model (1) is effective.

#### 4.1.2 Separate analysis

The regression results are shown in Table 5. Column (1) reports the regression results when putting the five dimensions into the model simultaneously. Both openness and conscientiousness have a significant positive impact on the reemployment of retirees, with coefficients of 0.151 and 0.362, respectively. The effects of the other three dimensions, extraversion, agreeableness, and emotional stability, are not significant. Columns (2) to (6) show the regression results for each of the five dimensions. Openness and conscientiousness are both significant at the 1% statistical level, with coefficients of 0.193 and 0.356, respectively. The other dimensions are not significant. The regression results of all dimensions show high goodness of fit, ranging from 0.2632 to 0.2728.

**Table 5:** Logit regression of the impact of five dimensions of non-cognitive skills on the reemployment of retirees.

	(1)	(2)	(3)	(4)	(5)	(6)
Variables	Reemployment of retirees					
Extraversion	−0.089	0.017				
	(0.056)	(0.057)				
	[−0.187, 0.036]	[−0.090, 0.138]				
Agreeableness	−0.081		0.028			
	(0.077)		(0.060)			
	[−0.236, 0.069]		[−0.093, 0.140]			
Openness	0.151**			0.193***		
	(0.063)			(0.056)		
	[0.022, 0.270]			[0.077, 0.299]		
Conscientiousness	0.362***				0.356***	
	(0.082)				(0.072)	
	[0.183, 0.505]				[0.203, 0.479]	
Emotional stability	−0.065					−0.071
	(0.048)					(0.047)
	[−0.153, 0.036]					[−0.155, 0.028]
Control variables	Yes	Yes	Yes	Yes	Yes	Yes
The fixed effects of different age groups	Yes	Yes	Yes	Yes	Yes	Yes
Pseudo R^2^	0.2728	0.2632	0.2632	0.2670	0.2697	0.2635
Observations	3,693	3,693	3,693	3,693	3,693	3,693

The standard errors of clustering robustness at the provincial level are shown in parentheses, and 95% confidence interval in square brackets. ∗*p* < 0.05, ∗∗*p* < 0.01, ∗∗∗*p* < 0.001.

### 4.2 Heterogeneous analysis

Gender, household registration, age and education are not only important factors influencing individuals’ decisions to act, but can also distinguish groups into distinct categories. Individuals in different groups face different opportunities and situations. First, a youthful bias in age identity may contribute to more employment opportunities for relatively young older adults. Second, men may have more energy to participate in the labor market compared to women. Third, urban older adults may have more employment opportunities and choices. Fourth, educational attainment is correlated with market demand, and the more educated older adults are, the more likely they are to be favored by employers.

Based on the above speculation, we conducted heterogeneity analysis. Table 6 shows the following: (1) non-cognitive skills contribute significantly to reemployment for retired men only, with a coefficient of 0.386; the effect on retired women is not significant. (2) Non-cognitive skills significantly impact the reemployment of both urban and rural retirees, and the effect is greater for the latter, with a coefficient of 0.399. (3) Compared with retirees over 70 years old, non-cognitive skills are more likely to affect the reemployment of younger retirees (65 and younger, 66–70 years old), with coefficients of 0.397 and 0.361, respectively. (4) The effect of non-cognitive skills on retirees with “No schooling” and with “primary school” degrees is more significant, with coefficients of 0.458 and 0.387, respectively. The R^2^ values were also used as a measure, and the goodness of fit of each model reached above 0.1320, indicating that non-cognitive skills explain reemployment in heterogeneous groups well.

**Table 6:** Heterogeneity analysis of the impact of non-cognitive skills on retiree reemployment.

	Gender		Household registration	
Variables	Male	Female	Urban	Rural
Non-cognitive skills	0.386***	0.311	0.256**	0.399***
	(0.117)	(0.204)	(0.118)	(0.137)
	[0.157, 0.615]	[−0.089, 0.712]	[0.025, 0.488]	[0.129, 0.668]
Control variables	Yes	Yes	Yes	Yes
The fixed effects of different age groups	Yes	Yes	Yes	Yes
Pseudo R^2^	0.3004	0.2317	0.1565	0.1320
Observations	2052	1,641	1,461	2,232

	Age (years)			
Variables	65 and below	66–70	71–75	76 and above
Non-cognitive skills	0.397***	0.361**	0.241	0.363
	(0.149)	(0.182)	(0.19)	0.331
	[0.105, 0.689]	[0.004, 0.718]	[−0.132, 0.614]	[−0.287, 1.013]
Control variables	Yes	Yes	Yes	Yes
The fixed effects of different age groups	Yes	Yes	Yes	Yes
Pseudo R^2^	0.2939	0.2309	0.2340	0.2359
Observations	1,391	1,176	698	421

	Education			
Variables	No schooling	Primary school	Junior high school	Senior high school and above
Non-cognitive skills	0.458***	0.387**	0.21	0.117
	(0.165)	(0.178)	(0.202)	(0.342)
	[0.134, 0.782]	[0.039, 0.735]	[−0.187, 0.607]	[−0.553, 0.787]
Control variables	Yes	Yes	Yes	Yes
The fixed effects of different age groups	Yes	Yes	Yes	Yes
Pseudo R^2^	0.1984	0.2845	0.3355	0.3159
Observations	1,520	875	785	511

The standard errors of clustering robustness at the provincial level are shown in parentheses, and 95% confidence interval in square brackets. ∗*p* < 0.05, ∗∗*p* < 0.01, ∗∗∗*p* < 0.001.

### 4.3 Mechanism analysis

First, we tested H2, that is, whether social capital has an impact on the reemployment of retirees. The following model (2) was constructed for this study.


(2) $$\text{Wor}k_{i}=\alpha_{2}+\lambda_{1}{\text{Capital}}_{i}+\gamma_{2}X_{i}+\zeta_{i}$$

In model (2), if the coefficient of social capital is significantly positive, then this means that social capital can promote the reemployment of retirees.

Second, we tested H3, that is, whether social capital plays an intermediary role. Referring to the intermediary effect test method of Wen and Ye (41), the following steps were applied in the study. First, model (1) was regressed to demonstrate the contribution of retirees’ non-cognitive skills to their reemployment. Second, model (3) was tested to prove the positive effect of non-cognitive skills on social capital. Finally, the intermediary role of social capital was demonstrated by putting both non-cognitive skills and social capital into model (4) for testing. The models are as follows:


(3) $${\text{Capital}}_{i}=\alpha_{3}+\beta_{2}{\text{Noncognitive}}_{i}+\gamma_{3}X_{i}+\mu_{i}$$

(4) $$\text{Wor}k_{i}=\alpha_{4}+\beta_{3}{\text{Noncognitive}}_{i}+\lambda_{2}{\text{Capital}}_{i}+\gamma_{4}X_{i}+\eta_{i}$$

${\text{Capital}}_{i}$ represents social capital, $\lambda_{1}$ and $\lambda_{2}$ are its correlation coefficients; $\alpha_{2}$, $\alpha_{3}$ and $\alpha_{4}$ are constant terms; $\beta_{2}$ and $\beta_{3}$ represent coefficients of non-cognitive skills; $\gamma_{2}$, $\gamma_{3}$ and $\gamma_{4}$ are correlation coefficients of control variables; $\zeta_{i}$, $\mu_{i}$ and $\eta_{i}$ are random error terms. The detailed test results are shown in Table 7.

**Table 7:** Mechanism analysis—social capital.

	(1)	(2)	(3)	(4)
Variables	Reemployment of retirees		Social capital	Reemployment of retirees
Non-cognitive skills	0.354***		0.540***	0.354***
	(0.098)		(0.153)	(0.103)
	[0.161, 0.547]		[0.225, 0.855]	[0.152, 0.555]
Social capital		0.037**		0.033**
		(0.015)		(0.016)
		[0.007, 0.067]		[0.002, 0.064]
Control variables	Yes	Yes	Yes	Yes
The fixed effects of different age groups	Yes	Yes	Yes	Yes
Pseudo R^2^	0.2683	0.2675	0.0707	0.2700
Observations	3,693	3,669	3,669	3,669

The standard errors of clustering robustness at the provincial level are shown in parentheses, and 95% confidence interval in square brackets. ∗*p* < 0.05, ∗∗*p* < 0.01, ∗∗∗*p* < 0.001.

Column (1) shows the benchmark regression result. The impact coefficient of the non-cognitive skills of the retirees on reemployment is 0.354, which is significant at 1%. The value of R^2^ is 0.2683. Column (2) shows the regression results of model (2). The impact coefficient of social capital on the reemployment of retirees is 0.037, which is significant at 5%. The value of R^2^ is 0.2675. H2 holds, which proves that the model (2) is set correctly. In other words, the accumulation of social capital can indeed promote the reemployment of Chinese retirees. Column (3) shows the regression results of model (3). The impact coefficient of non-cognitive skills on social capital is 0.540, which is significant at 1%. But the value of R^2^ is only 0.0707. Column (4) shows the regression result of model (4). The coefficient of non-cognitive skills is 0.354, and the coefficient of social capital is 0.033, which is significant at the level of 1 and 5%, respectively. The value of R^2^ is 0.2700. H3 is thus validated, which shows that the intermediary effect model (i.e., model (3) (4)) is set correctly, and non-cognitive skills can promote the reemployment of Chinese retirees through the accumulation of social capital. In addition, according to the above results, the indirect effect of non-cognitive skills on reemployment through social capital is $\beta_{2}*\lambda_{2}$ = 0.018, and its proportion of the total effect (effect size) is $\beta_{2}*\lambda_{2}/\beta_{1}$ = 5.03%. This result indicates that the contribution of non-cognitive skills to reemployment is indeed partly through the accumulation of social capital, and the contribution of reemployment through social capital accounts for 5.03% of the total effect.

## 5 Discussion

### 5.1 Research findings

Firstly, this study reflects that non-cognitive skills can significantly promote the reemployment of retirees. The results remained stable after controlling macro factors. Among the five dimensions of non-cognitive skills, conscientiousness and openness have the more significant effect on reemployment. In general, organized, strong-willed, and reliable individuals tend to value wealth accumulation (42). Imaginative, creative, and unconventional individuals are more sensitive to job information when seeking employment, and more tolerant and adaptable to different careers. As a result, retirees who are open and conscientious are more likely to be reemployed. The above findings are consistent with those of Cuesta and Budría (43).

Secondly, the influence of non-cognitive skills on the reemployment of retirees is significantly different among different retired groups. It is highly related to gender, household registration, age, and education level. Notably, this study found that: (1) non-cognitive skills only play significant influential roles on the reemployment of male retirees. This result is contrary to previous conclusions in research. For example, Bennett et al. (44) assumed that there was no significant relationship between traditional gender roles and reemployment after retirement. The different conclusions may be determined by cultural differences. Gender norms such as “men dominate outside the home while women dominate within it” and “men are superior to women” have been a long-standing cultural tradition in China. Compared with women, Chinese men have extensive social networks and more social capital. They have more opportunities to improve their non-cognitive skills, which in turn promotes opportunities for reemployment. (2) Non-cognitive skills contribute more to the reemployment of rural retirees than those from urban regions. The rationale for this finding may lie in the dualistic economic structure of urban and rural areas in China (17). Compared with urban areas, the standard of living in rural China is relatively backward. Given concerns around livelihood, the rural older adults usually remain in the labor force until they lose the ability to work. (3) The influence of non-cognitive skills on reemployment decreases as retirees age. As they get older, their ability to work and their willingness to reenter the labor force weaken gradually, and reemployment possibilities also decrease (45). (4) Non-cognitive skills have greater impacts on the reemployment of retirees with lower education levels. Educational attainment is an important component of traditional human capital. However, Non-cognitive skills such as self-confidence and ambition can effectively and positively affect work skills and experiences, thus increasing the benefits of work skills and experience (46), and effectively promoting the reemployment of retirees with less education.

Thirdly, social capital plays an intermediary role. Non-cognitive skills can increase the social capital of retirees, which in turn will help encourage their reemployment. This is because retirees with greater non-cognitive skills usually have relatively strong interpersonal skills. Simultaneously, they are also able to increase their social networks and use their connections to acquire support for reentry into the job market. Social capital therefore provides retirees with a broader selection of career opportunities (47).

### 5.2 Policy implications

The findings of this study have useful policy implications. In terms of cultivating and promoting retirees’ non-cognitive skills, we propose that the government consider offering continuing education through lectures and courses available to the older adults. At the same time, the government should provide learning opportunities for the rural older adults, for example, by building libraries in the countryside. Secondly, the government should encourage retirees to leave their homes and actively participate in social activities, by improving transportation facilities and community living environment. This will encourage retirees to broaden their social networks, accumulate social capital, and obtain more job information and emotional support through social interaction.

In relation to policy implications for the protection and rights of retirees seeking reemployment. Firstly, the government should improve public employment services, through actions like establishing unemployment benefits for the older adults, improving employment injury insurance based on the physiological characteristics of older workers, and providing reemployment assistance through various means. Secondly, the government should strengthen legislation on employment protection for the older adults, in addition to encouraging enterprises to hire older workers through tax incentives and financial subsidies. They should also strive to eliminate employment discrimination. Finally, enterprises should increase the number of employment positions and training opportunities that are suitable for the older adults, while also addressing age-specific issues such as working hours, intensity of labor, and welfare benefits.

### 5.3 Theoretical contributions

This study offers three theoretical implications for the study of retiree reemployment.

First, the study supports its hypothesis related to the impact of non-cognitive skills on the reemployment of retirees. This theoretical framework can be applied in future studies on the effect of human capital on reemployment. Second, we addressed a gap in the literature by comparing groups of retirees based on gender, household registration, age, and education level (48, 49). These findings provide new theoretical evidence and insights for future research on diverse retirees. Third, this study found that social capital is important to the promotion of reemployment for this age group, especially in a nepotist society like China. In sum, the framework of this study is helpful to provide theoretical reference for future research on the reemployment of retirees.

### 5.4 Conclusions and limitations

Using the BFI, we measured the non-cognitive skills of Chinese retirees. We also developed a theoretical analysis framework on non-cognitive skills and reemployment of retirees, and we used CFPS to empirically test the effect of non-cognitive skills on the reemployment of retirees. The study found that it significantly helped, with traits such as openness and conscientiousness playing the most significant role. Grouped regressions show that non-cognitive skills are strongly related to individual characteristics, and its connection to reemployment is greater for retirees who are male, have a rural household registration, are younger, and are less educated. Through the intermediary effect test of social capital, this study found that improving non-cognitive skills can help retirees accumulate social capital, thus impacting the possibility for reemployment.

This study also has some limitations. First, it only used the cross-sectional data of CFPS in 2018, which may lead to a certain degree of sample deviation. Secondly, due to the ambiguity of social capital, academics have not yet developed a suitable unified measurement index. Putnam (50) measured social capital from three lenses, social network, trust, and reciprocity norms, while Horng and Wu (51) divided the concept into bonding social capital and bridging social capital. Different metrics may lead to different conclusions. In addition, this empirical research is grounded in Chinese data. Subsequent studies can be conducted in other countries to verify the universality of the research results.

## Data availability statement

Publicly available datasets were analyzed in this study. This data can be found here: www.isss.pku.edu.cn/cfps/.

## Ethics statement

The studies involving human participants were reviewed and approved by the Biomedical Ethics Committee of Peking University (IRB00001052-14010). The patients/participants provided their written informed consent to participate in this study. The studies were conducted in accordance with the local legislation and institutional requirements. The participants provided their written informed consent to participate in this study.

## Author contributions

XS presented the ideas for this manuscript, developed the methodology, wrote the theoretical analysis, results, discussions, and conclusions. HJ and JW provided theoretical guidance throughout the process, grasped the overall writing direction, and revised and edited the entire manuscript. HS provided methodological guidance and reviewed and edited the content. All authors have read and agree to the published version of the manuscript.

## Footnotes

^1^ http://www.stats.gov.cn/tjsj/ndsj/2019/indexch.htm
