# Supplementary material for: How do the non-cognitive skills affect retirees’ reemployment? Evidence from China
Source: Front Public Health. 2023 Dec 19;11:1128241. doi: 10.3389/fpubh.2023.1128241 (PMC10758448; doi:10.3389/fpubh.2023.1128241)
Supplement: Supplementary file 1 [file Table_1.DOCX]

Appendix A Interpretation of BFI.

| **Dimension** | **Interpretation** | **Corresponding items in CFPS** |
| --- | --- | --- |
| Extraversion | Sociable, warm, talkative, assertive, cheerful | "outgoing and sociable", "talkative", "reserved and conservative" |
| Agreeableness | Altruistic, trustworthy, modest, cooperative | "considerate of others", "tolerant by nature", and "rude to others" |
| Openness | Imaginative, creative, unconventional, emotionally, artistically sensitive | "originality", "attach importance to artistic and aesthetic experience", and "rich imagination" |
| Conscientiousness | Organized, strong-willed, persistent, reliable, effective | "efficient", "rigorous and serious", and "often lazy" |
| Emotional stability | Calm and relaxed, able to handle stress well | "often worried", "easily nervous" and "copes well with stress" |

Appendix B Correlation between indicators.

| **Five dimensions** | **Secondary indicators** | **(1)** | **(2)** | **(3)** | **(4)** | **(5)** | **(6)** | **(7)** | **(8)** | **(9)** | **(10)** | **(11)** | **(12)** | **(13)** | **(14)** | **(15)** |
| --- | --- | --- | --- | --- | --- | --- | --- | --- | --- | --- | --- | --- | --- | --- | --- | --- |
| Extraversion | (1) Talkative | 1.000 |  |  |  |  |  |  |  |  |  |  |  |  |  |  |
|  | (2) Outgoing and sociable | 0.347* | 1.000 |  |  |  |  |  |  |  |  |  |  |  |  |  |
|  | (3) Reserved and conservative (reversed) | 0.004 | -0.007 | 1.000 |  |  |  |  |  |  |  |  |  |  |  |  |
| Agreeableness | (4) Considerate of others | 0.156* | 0.258* | -0.075* | 1.000 |  |  |  |  |  |  |  |  |  |  |  |
|  | (5) Tolerant by nature | 0.176* | 0.282* | -0.037 | 0.317* | 1.000 |  |  |  |  |  |  |  |  |  |  |
|  | (6) Rude to others (reversed) | -0.145* | -0.069* | 0.115* | 0.037 | 0.047* | 1.000 |  |  |  |  |  |  |  |  |  |
| Openness | (7) Rich imagination | 0.223* | 0.245* | -0.133* | 0.263* | 0.183* | -0.108* | 1.000 |  |  |  |  |  |  |  |  |
|  | (8) Attach importance to artistic and aesthetic experience | 0.194* | 0.279* | -0.118* | 0.170* | 0.137* | -0.096* | 0.364* | 1.000 |  |  |  |  |  |  |  |
|  | (9) Originality | 0.231* | 0.190* | -0.099* | 0.102* | 0.125* | -0.187* | 0.380* | 0.320* | 1.000 |  |  |  |  |  |  |
| Conscientiousness | (10) Efficient | 0.225* | 0.283* | -0.088* | 0.252* | 0.232* | -0.058* | 0.324* | 0.259* | 0.260* | 1.000 |  |  |  |  |  |
|  | (11) Often lazy (reversed) | 0.031 | 0.071* | 0.160* | 0.083* | 0.102* | 0.205* | -0.031 | -0.037 | -0.015 | 0.157* | 1.000 |  |  |  |  |
|  | (12) Rigorous and serious | 0.181* | 0.189* | -0.012 | 0.200* | 0.251* | 0.008 | 0.199* | 0.147* | 0.169* | 0.244* | 0.134* | 1.000 |  |  |  |
| Emotional stability | (13) Copes well with stress | 0.156* | 0.237* | -0.042 | 0.204* | 0.233* | -0.052* | 0.277* | 0.196* | 0.188* | 0.237* | -0.021 | 0.124* | 1.000 |  |  |
|  | (14) Easily nervous (reversed) | -0.054* | 0.020 | 0.215* | -0.066* | -0.018 | 0.151* | -0.098* | -0.090* | -0.097* | -0.064* | 0.198* | -0.034 | 0.008 | 1.000 |  |
|  | (15) Often worried (reversed) | -0.099* | -0.044* | 0.180* | -0.108* | -0.078* | 0.169* | -0.134* | -0.103* | -0.182* | -0.131* | 0.068* | -0.066* | -0.021 | 0.373* | 1.000 |

Note: * p<0.01
